# Inhibition of Oxidative Stress-Induced Ferroptosis Can Alleviate Rheumatoid Arthritis in Human

**DOI:** 10.1155/2024/9943747

**Published:** 2024-02-26

**Authors:** Yang Liu, Jiang Liang, Zongge Sha, Changfu Yang

**Affiliations:** ^1^School of Basic Medicine, Guizhou University of Traditional Chinese Medicine, Guiyang 550025, China; ^2^First Affiliated Hospital, Guizhou University of Traditional Chinese Medicine, Guiyang 550025, China

## Abstract

Rheumatoid arthritis (RA) is a chronic autoimmunity illness, mainly featured with synovitis of the joint. The specificity of ferroptosis is disparate in different diseases, and the mechanism of ferroptosis in RA has some uncertainty yet. Therefore, the mechanism of ferroptosis was deeply observed in RA patients and animal models. In this paper, plasma of RA patients, the tumor necrosis factor-alpha-induced human synovial fibroblasts, and an animal model of arthritis induced by collagen were applied to initially inquire about the therapeutic effect of ferroptosis. For the RA patients, ELISA detected protein expression of glutathione (GSH), GPX4, Nrf2, Keap-1, and ferritin. In cell experiments, erastin or fer-1 regulated the invasion of human synovial fibroblast cells, mitochondrial membrane potential, reactive oxygen species (ROS) expression, marker protein, and so on. For the animal experiments, 32 Sprague–Dawley male rats were randomly separated into four groups with a collagen-induced RA model for 14 days and administered with erastin or fer-1 for 35 days. The expressions of GSH, GPX4, Nrf2, and Keap-1 were lower, and the ferritin was higher in RA patients, and the expressions of these proteins varied significantly after disease remission. In addition, ferroptosis inactivation also reduced the proliferation and migration ability, mitochondrial membrane potential, and ROS in cells. We discovered unexpectedly that activation of ferroptosis meaningfully forbore the foot swelling in animals with CIA, reduced arthritis scores, destruction of bone, and articular synovitis, and also decreased the high expression of inflammatory factors in plasma. There is a nonlinear relationship between human disease manifestations and animal model pathology. Ferroptosis regulating in RA for humans or animals may produce different effects.

## 1. Introduction

The clinical manifestations of rheumatoid arthritis (RA) are morning stiffness, symmetrical polyarticular swelling and pain, joint stiffness, and even deformation. The basic pathological changes of RA are pannus formation and synovial hyperplasia, resulting in the damage and erosion of subchondral bone and articular cartilage, with a high teratogenic rate, which greatly influences the well-being of patients [1]. The pathogenesis of RA is still uncertain. As Rudolf Virchow said in the 19th century, diseases do not exist independently, and they are the process of continuing life under changed conditions. The link between pathological development and clinical manifestations is the mechanism of disease. In order to maintain their own survival process, organisms need to constantly exchange materials with the external environment and stabilize their own internal environment, which involves a lot of intercellular mechanism adjustment [2]. A variety of intracellular organelles have their own redox poise; in cells, one of the main sources of reactive oxygen species (ROS) is mitochondria. ATP synthesis produces ROS during oxygen metabolism, and ROS is thought to be a byproduct of ATP metabolism [3]. Cells are the basic organizational units of biological systems, and the mechanisms controlling cell differentiation, development, propagation, and death are critical to learning the function of life and the process of disease development on Earth [4]. Ferroptosis is a specialized mode of cell death that is diverse from conventional cell death, such as apoptosis or autophagic necrosis [5]. Ferroptosis is mainly the imbalance between intracellular ROS generation and degradation, affects glutathione (GSH) peroxidase activity directly or indirectly, and reduces cell antioxidant capacity, accompanied by a large accumulation of iron, which causes the Fenton reaction, cause cell oxidative death, namely ferroptosis [6]. Existing basic studies of RA have confirmed that lymphocytes, monocytes, macrophages, and B cells proliferate abnormally and accumulate around the joint synovium [7, 8]. At the same time, the local microenvironment of the diseased synovial membrane is under oxidative stress, in which functional confusion between peroxides and the oxidation resistance system is more conducive to enhance the high expression of NF-*κ*B in RA [9, 10]. The regulatory manifestation of ferroptosis in the etiopathogenesis of RA has uncertainty. Therefore, the paper aims to examine the role of the ferroptosis mechanism in RA.

## 2. Materials and Methods

### 2.1. Chemicals and Reagents

Erastin and ferrostatin-1 (fer-1) were acquired from Selleck (Lot: s7242 and s7243, Shanghai, China). *β*-Actin, IRP2, and VDAC rabbit mAb were received from CST (Lot: #4970, #37135, and #4661, Shanghai, China). Rabbit anti-DMT1, anti-TFR2, and anti-GPX4 antibody were ordered from Bioss (Lot: bs-3577R, bs-9894R, and bs-3884R, Beijing, China). ROS detection kit, JC-1, 2-(4-amidinophenyl)-6-indolecarbamidine dihydrochloride (DAPI), GSH, and GSSG assay kit were derived from Beyotime Biotechnology (Lot: S0033S, C2003S, C1005, and S0053, Shanghai, China). A hematoxylin–eosin (HE) staining kit was obtained from Phygene (Lot:210524, Fujian, China). ELISA kit for ferritin and ELISA kits for glutathione peroxidase 4 (GPX4) were ordered from Cloud-Clone Corp (Lot: SEA518Hu and L220512118, Wuhan, China). Human nuclear factor erythroid 2-related factor 2 (Nrf2) ELISA kit and Human kelch-like ECH-associated protein 1 (Keap1) ELISA kit were purchased from CUSABIO (Lot: S11025461 and S04025460, Wuhan, China). Eponate 12™ embedding kit was purchased from TED PELLA (Lot: 18010, Guangzhou, China). Uranyl acetate, lead citrate trihydrate, and OsO_4_ were purchased from Sigma (Lot: U25690, 15326, and O5500, Hongkong, China).

### 2.2. Redox Reactions in RA Patients

RA patients aged 18–65 years were collected according to the diagnostic criteria established by ACR/EU-LAR, excluding other autoimmune diseases, cardiovascular and cerebrovascular diseases, abnormal liver and kidney functions, history of surgery, history of smoking, and pregnant and lactating women. RA patients were divided into the remission phase (10 men and 10 women patients, DAS28 < 2.6) and the active phase (10 men and 10 women patients, DAS28 > 5.1). Healthy people aged 18–65 were collected as controls (10 males and 10 females). Peripheral venous blood was collected on fasting, and plasma was collected to detect GSH, GPX4, Nrf2, Keap1, and ferritin by ELISA. The research was authorized by the Ethics Review Committee of the First Affiliated Hospital of Guizhou University of Traditional Chinese Medicine (reference number: K2022-1).

### 2.3. Culture Cell

The human fibroblast-like synoviocytes were derived from the synovial membrane of the joint in patients with RA. Cells were grown in DMEM high-glucose complete medium at 37°C and 5% CO_2_, respectively.

### 2.4. Cellular Proliferation

Human fibroblast-like synoviocytes (2 × 10^3^ cells) were blended in 96-well plates. Normal, control, and erastin or fer-1 groups were established. Different concentrations of erastin (0, 18.28, 36.56, 73.12, 109.68, 146.24, and 182.80 *µ*M) or fer-1 (0, 38.12, 76.23, 152.47, 228.70, 304.94, and 381.17 *µ*M). CCK-8 (10 *µ*L) was mixed into each well for 48 hr. After 4 hr incubation, samples were monitored absorbance at 450 nm with a Thermo Scientific microplate reader (Shanghai, China).

In addition, human fibroblast-like synoviocytes (2 × 10^3^ cells) were merged in 96-well plates. The culture medium was altered after 24 hr, and control and erastin (18.28, 36.56, and 73.12 *µ*M) or fer-1 (76.23, 152.47, and 228.70 *µ*M) groups were fit up. Tumor necrosis factor-alpha (TNF-*α*) 20 *µ*g/L and erastin or fer-1 of different concentrations were added in triplicate. About 10 *µ*L CCK-8 was put in each well of every group after 48 hr proliferation at 37°C and 5% CO_2_. Monitoring absorbance at 450 nm by Thermo Scientific microplate reader after 4 hr incubation (Shanghai, China).

### 2.5. Wound-Healing Assay

Six-well plates were used for incubation after fusion of human fibroblast-like synoviocytes and 10% FBS with DMEM medium. Direct scratched or damaged cells were made with a width of 0.2–0.4 mm with a sterile pipette tip. Add all kinds of drugs and incubate for 48 hr. Migration and analysis of randomly selected cells on artificial wounds using Leica DMI 4000B (Buffalo Grove, USA).

### 2.6. Mitochondrial Membrane Potential and ROS Analysis

After the fusion of human fibroblast-like synoviocytes (4 × 10^4^ cells) and culture medium, six-well plates were used for incubation. Cells were cotreated with the erastin or fer-1 coalescing TNF-*α* (20 *µ*g/L) for 48 hr. JC-1 staining 1 mL or 2′,7′-dichlorodihydrofluorescein diacetate (DCFH-DA) was filled into each well. Cells were grown in 37°C surroundings for 20 min. Rinsed twice by staining buffer (1×) and observed under Cossim FR-4A fluorescence microscope (Beijing, China) or Thermo scientific fluorescence microplate reader (Shanghai, China).

### 2.7. Immunofluorescence Staining Analysis

Slides of cells were prepared to affiliate cells (3 × 10^3^ cells) in a 24-well plate. Cells were coalesced with the erastin or fer-1, accompanied by TNF-*α* (20 *µ*g/L) for 48 hr. Cleaning with PBS thrice after paraformaldehyde (4%) stayed at the well for 30 min. Overall, 5% goat serum was incubated for 1 hr, added 1 : 100 GPX4, and further incubated at 4°C overnight. Mixing rabbit antibody 1 : 300 in the dark for 2 hr to wash with PBS thrice, DAPI stained for 10 min at room temperature. Three visual fields were selected randomly from each hole to take photos with the Cossim FR-4A fluorescence microscope (Beijing, China) and analyzed.

### 2.8. Western Blot Analysis

Samples make use of columnar centrifugation and are quantified by BCA to extract total protein. Preparing separation gel (12%) and concentration gel (5%) to electrophorese. Different proportions of antibodies, including *β*-actin (1 : 6,000), IRP2 (1 : 1,000), TFR2 (1 : 1,000), VDAC (1 : 1,000), DMT1 (1 : 1,000), GPX4 (1 : 1,000), II antibody (1 : 5,000) were used.

### 2.9. Transmission Electron Microscopy Analysis

The samples were fixed by 3% glutaraldehyde and then fixed by 1% OsO_4_ with acetone and dehydrated step by step. The dehydrated samples were sequentially made into 50 nm thick ultrathin sections with a dehydrating agent and epoxy resin and then stained by uranyl acetate for 15 min and trihydrate lead citrate for 2 min. The changes in the mitochondrial membrane were observed under the FEI Tecnai F20 TEM microscope (Tokyo, Japan).

### 2.10. Animals

Sprague–Dawley (SD) male rats, specifically pathogen-free, 180–220 g and 6–8 weeks, were ordered from TengXin Biotechnology Co. Ltd., Chongqing, China, and bred by the animal experiment center of Guizhou University of Traditional Chinese Medicine. The Animal Ethics Committee of the Guizhou University of Traditional Chinese Medicine approved the experiment plan (reference number: 20210034).

### 2.11. Collagen-Induced Arthritis (CIA)

Rats fell casually into the control (*n* = 8) and model group (*n* = 24). Refer to the previous method [11]. After successful CIA model induction, the rats were randomly split into groups of a model, erastin 10 mg/kg and fer-1 10 mg/kg, which included eight rats in each group. The rats received drug intraperitoneal injection treatment for 35 consecutive days. Arthritis index scores, animal weight, and foot swelling of rats in each group were measured. Isoflurane gas anesthesia euthanized animals and drew its materials on the last day of the experiment.

### 2.12. Pathological Analysis of Synovium

Synovium was immersed in ethanol gradient concentration for 2 hr. The synovium was dipped in xylene for 30 min and in liquid paraffin for 15 min. The slides of a sample with 4 *µ*m thickness were dewaxed for 30 min. Slides were steeped in alcohol 10 min gradient elution, distilled water wash it for 1 min, stained with hematoxylin for 10 min and eosin for 1 min, steeped in gradient alcohol, and entered xylene lastly for 10 min and scanned by the Olympus CX43 microscope (Tokyo, Japan).

### 2.13. ELISA Assay for Plasma

According to the kit instructions, the standard and sample were added in wells to incubate. Further add the working solution for color development, plasma expression of MCP-1*α*, IL-17, IL-1*β*, IL-6, TNF-*α*, and CXCL-1 was measured.

### 2.14. Statistical Analysis

All experimental data were represented as mean ± SD. GraphPad Prism 9.0 software was used to data analysis based on non-parametric rank sum test. A value of *P* < 0.05 was considered significant.

## 3. Results

### 3.1. Oxidative Stress Transition

**Figure 1:**
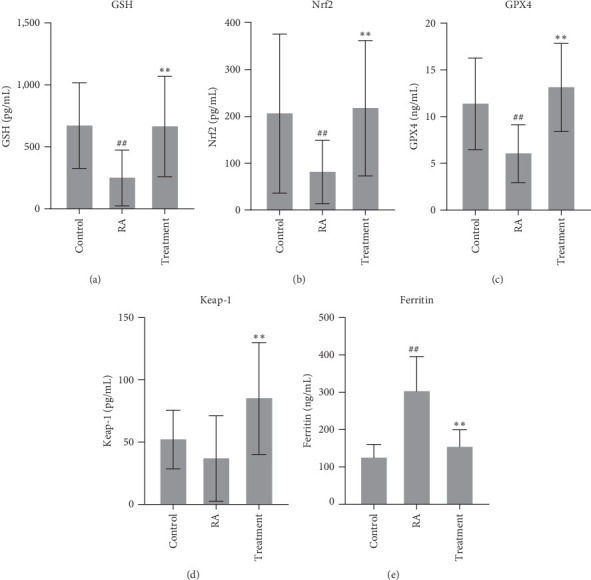
GSH, GPX4, Nrf2, Keap1, and ferritin expressions in plasma were identified by ELISA (a–e). After treatment, ferritin was decreased significantly, while GSH, GPX4, Nrf2, and Keap1, which are related to oxidative stress, were increased (^##^*P* < 0.01 vs. Ctrl group; *⁣*^*∗∗*^*P* < 0.01 vs. RA group, *n* = 20).

About antioxidant protein for GSH, GPX4, Nrf2, and Keap1 expressions in plasma were identified by ELISA, which were upregulated in the healthy group and the treatment group, and downregulated in the patients with RA. From these results, we can see the overall improvement of the antioxidant capacity in patients before and after treatment. Compared with healthy people, the level of ferritin in patients increased significantly before treatment and decreased significantly after treatment, implying that ferritin has participated in the development degree of disease (Figure 1).

### 3.2. Inhibition of Ferroptosis Lessens Proliferation in Human Cells

**Figure 2:**
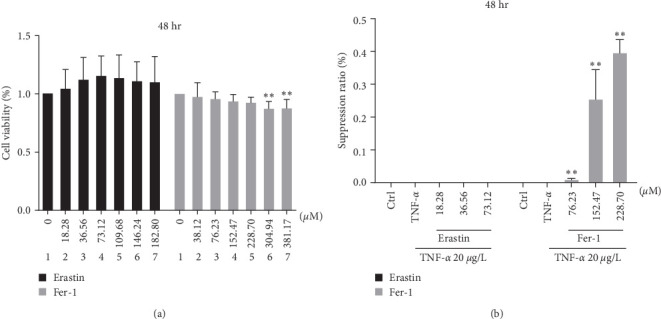
Ferroptosis influenced the development and suppression ratio of synoviocytes. Concentrations gradient of erastin or fer-1 were added to the cells and incubated for 48 hr. About 20 *μ*g/L TNF-*α* and different concentrations of erastin or fer-1 were added in fibroblast-like synoviocytes to coculture and promoted their proliferation, and the inhibition rate of erastin or fer-1 on cells was analyzed: (a) cell viability of synoviocyte; (b) the suppression ratio of synoviocyte (*⁣*^*∗∗*^*P* < 0.01 vs. TNF-*α* group, *n* = 3).

The optimal concentration of the drug was gained since 48 hr coincubation of synoviocytes and drug. In the light of optimal concentration, the activator erastin (18.28, 36.56, and 73.12 *µ*M) or inhibiting agent fer-1 (76.23, 152.47, and 228.70 *µ*M) concentrations to be administered in subsequent experiments were determined. After determining the concentration of erastin and fer-1, further analysis of erastin or fer-1 inhibitory effects on cells concluded that erastin did not affect cell activity, while fer-1 significantly inhibited cell growth (Figures 2(a) and 2(b)).

### 3.3. Inhibition of Ferroptosis Reduces Human Cells' Migration or Growth Ability

**Figure 3:**
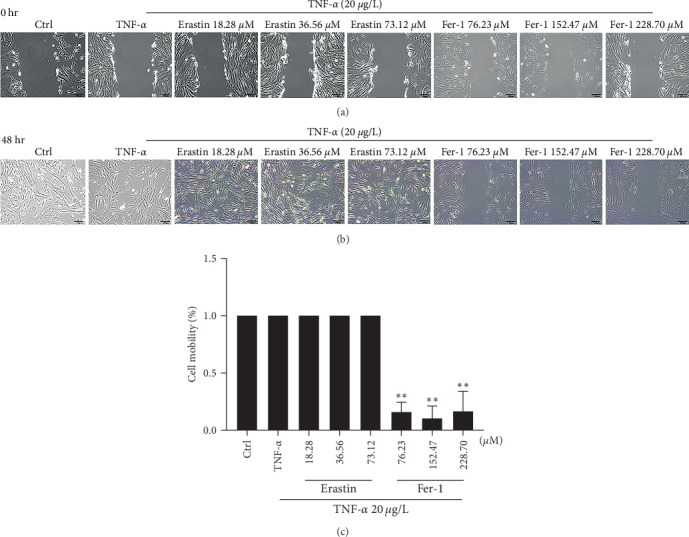
The in vitro wound healing assay of ferroptosis on synoviocytes. TNF-*α* 20 *μ*g/L and different concentrations of erastin or fer-1 were, respectively, added in cells to coculture for 48 hr: (a) areas of cell wound between groups; (b) areas of cell repair between groups; (c) cell mobility results (*⁣*^*∗∗*^*P* < 0.01 vs. TNF-*α* group, *n* = 3).

Cell migration is one of the basic functions of normal cells, and it is also a universal form of movement. An inflammatory response is closely related to cell migration. The human fibroblast-like synoviocytes were incubated for 48 hr, and cell migration ability did not change in the control group and TNF-*α* group. Compared with the TNF-*α* group, different concentrations of erastin or fer-1 could significantly affect the migration ability of human fibroblast-like synoviocytes. Inhibition of iron death signal can significantly reduce human cells migration ability (Figure 3(a)–3(c)).

### 3.4. Inhibition of Ferroptosis Restrains Mitochondrial Membrane Potential and ROS Release in Human Cell

**Figure 4:**
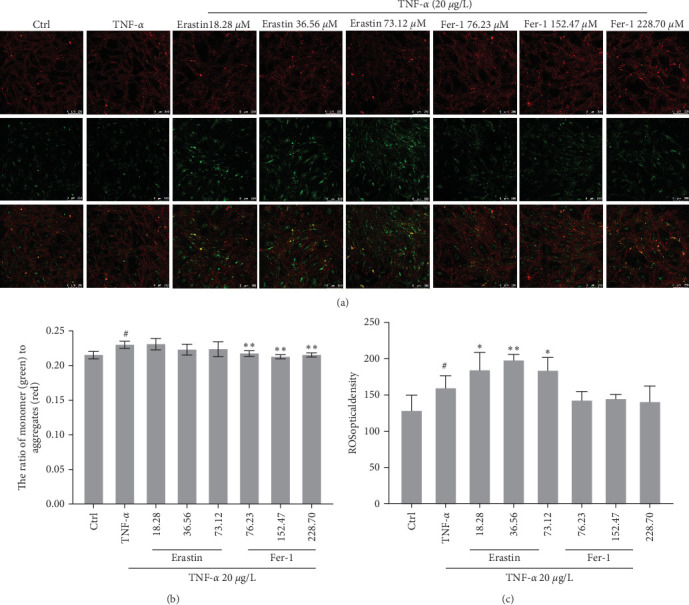
Ferroptosis impact on mitochondrial membrane potential and reactive oxygen species (ROS) in synoviocytes. About 20 *μ*g/L TNF-*α* and concentrations gradient of erastin or fer-1 were respectively added in cells to coculture for 48 hr: (a and b) JC-1 company with high mitochondrial membrane potential to accumulate a polymer and produce red fluorescence in the mitochondria matrix. JC-1 cannot aggregate when the mitochondrial membrane potential is lower in the mitochondria matrix. Meanwhile, JC-1 can produce green fluorescence, which facilitates the detection of changes in mitochondrial membrane potential by changes in fluorescence color. It is usual to use the ratio of red–green fluorescence to measure the ratio of mitochondrial depolarization; (c) intracellular ROS was monitored by fluorescence spectrophotometer (^#^*P* < 0.05 vs. Ctrl group; *⁣*^*∗*^*P* < 0.05 and *⁣*^*∗∗*^*P* < 0.01 vs. TNF-*α* group, *n* = 5).

In Figures 4(a) and 4(b), after coincubation with erastin, the mitochondrial membrane potential in cells was significantly enhanced and showed bright green fluorescence. But coincubation with fer-1, the mitochondrial membrane potential in cells was decreased and showed obvious red fluorescence. In Figure 4(c), different concentrations of erastin or fer-1 could significantly heighten or reduce ROS release in human fibroblast-like synoviocytes.

### 3.5. Inhibition of Ferroptosis Enhances Expression of GPX4 in Human Cells

**Figure 5:**
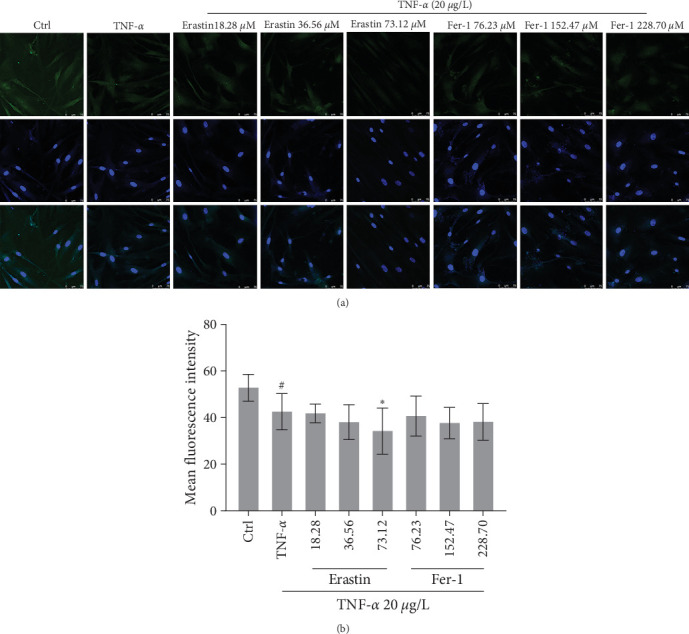
Effects of ferroptosis on GPX4 in synoviocytes. About 20 *μ*g/L TNF-*α* and different concentrations of erastin or fer-1 were, respectively, added in cells to co-culture for 48 hr: (a) fluorescent images of cells; (b) GPX4 expression was evaluated by mean fluorescence intensity (^#^*P* < 0.05 vs. Ctrl group; *⁣*^*∗*^*P* < 0.05 vs. TNF-*α* group, *n* = 5).

GPX4 protein was highly expressed in the control group. To compare with the control group, GPX4 in TNF-*α* declined significantly. After different concentrations of erastin or fer-1 were, respectively, added in cells, the antioxidant molecule GPX4 expression was inhibited by the highest dosage of erastin (Figure 5).

### 3.6. Ferroptosis Signaling in Human Fibroblast-Like Synoviocytes

**Figure 6:**
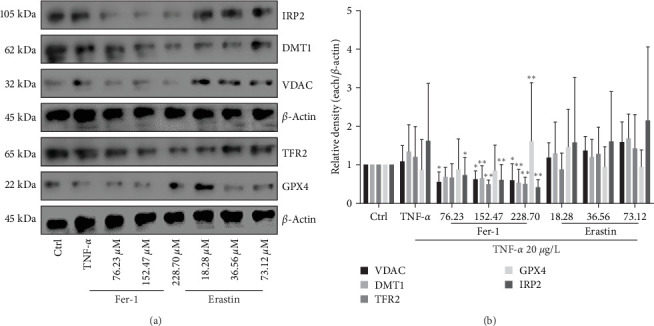
Effects of ferroptosis on VDAC, DMT1, TFR2, GPX4, and IRP2 in synoviocytes. About 20 *μ*g/L TNF-*α* and gradient concentration of erastin or fer-1 were, respectively, added in cells to coculture for 48 hr: (a) western blot image; (b) western blot gray values (*⁣*^*∗*^*P* < 0.05 and *⁣*^*∗∗*^*P* < 0.01 vs. TNF-*α* group, *n* = 5).

Erastin or fer-1 was an activator or inhibitor for ferroptosis; they added in cells could significantly regulate these key proteins of VDAC, DMT1, TFR2, GPX4, and IRP2 different expression. From these results, we can see that the activator can significantly activate the expression of VDAC, DMT1, TFR2, and IRP2 in the iron death signal, while the inhibitor can increase the level of the antioxidant molecule GPX4 (Figures 6(a) and 6(b)).

### 3.7. Effects of Inhibition of Ferroptosis on Mitochondrion

**Figure 7:**
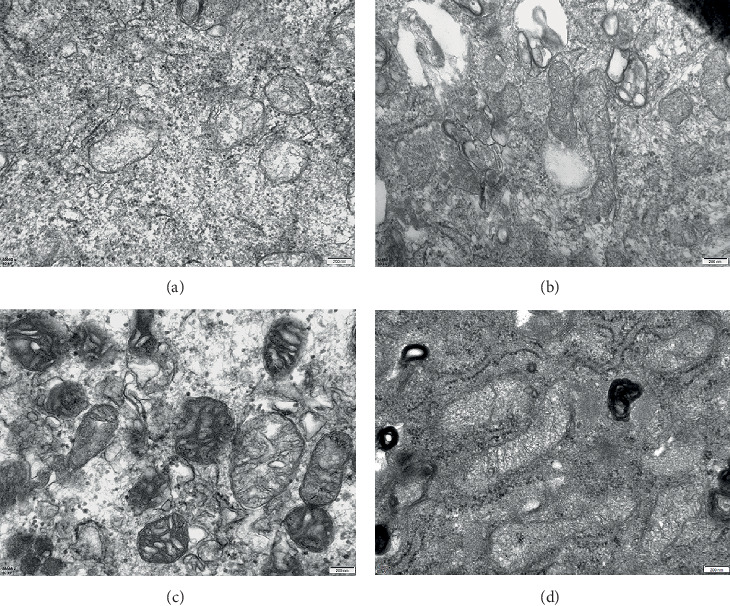
(a) In the control group, the mitochondria cristae was narrow and slender, and the density of some mitochondria matrix was slightly decreased; (b) mitochondria in the TNF-*α* group showed the shape became longer, the number increased, and the density of membrane increased; (c) in the fer-1 group, there were voids in the mitochondrion, but the mitochondrion shape became shorter, and the gap became wider, and part of the cristae was vesicular; (d) cavernous structure and membrane dense agglutination were shown in mitochondria of erastin group. The magnification of the above images is 20,000.

Erastin or fer-1 added in human fibroblast-like synoviocytes could significantly induce mitochondrion morphological change (Figure 7).

### 3.8. Ferroptosis Decreases Joint Inflammation

**Figure 8:**
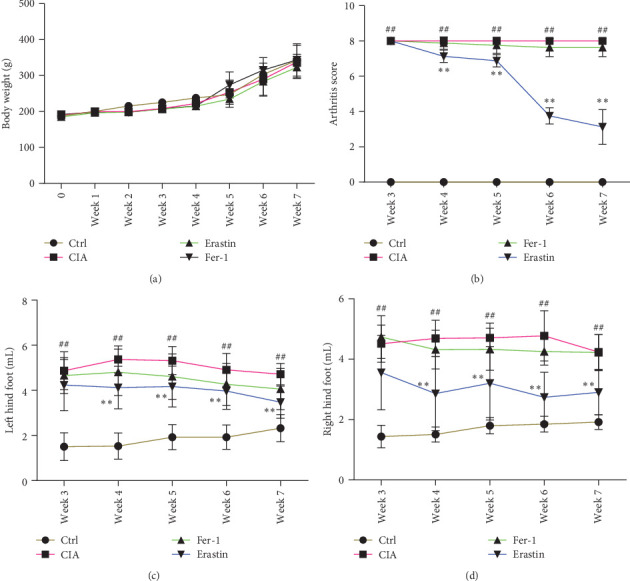
Ferroptosis affected the growth of animals and inflammatory swelling of feet. In total, 32 male SD rats were randomly grouped into four groups: Ctrl, CIA, CIA-Erastin, and CIA-Fer1. After the success of the CIA animal model, accepting erastin or fer-1 to stimulate continuously for 5 weeks. Body weight and arthritis scores were recorded once a week. The level of paw swelling was measured by foot volume measuring instrument once a week: (a) the growth curve of rats; (b) the severity of arthritis by the arthritis score; (c) the left hind foot; (d) the right hind foot (^##^*P* < 0.01 vs. Ctrl group and *⁣*^*∗∗*^*P* < 0.01 vs. CIA group, *n* = 8).

Results show the impact of ferroptosis on animal weight and arthritis scores. No significant difference was found in body weight between groups after 35 days (Figure 8(a)). Arthritis scores remained higher in the CIA and fer-1 groups compared to the control group. Erastin could significantly reduce the arthritis score induced by collagen in rats (Figure 8(b)). Fer-1 as the inhibitor did not limit the inflammation of CIA rats, but erastin as the activator could significantly restrict the inflammatory swelling of CIA rats (Figures 8(c) and 8(d)).

### 3.9. Ferroptosis Rescues Bone Tissue and Synovium in Animal Model

**Figure 9:**
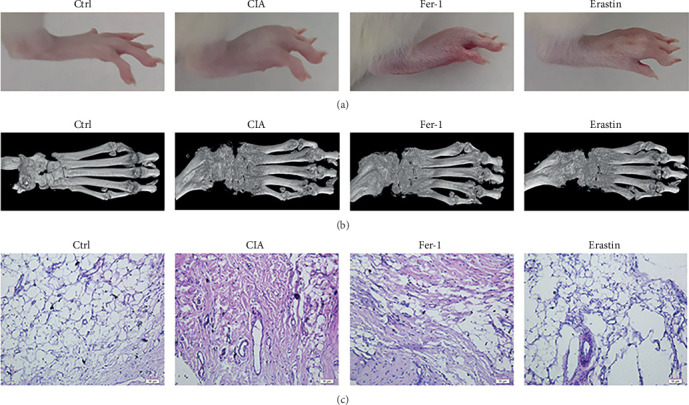
Ferroptosis interferes in animal bone tissue and synovium: (a) in comparison with the control group, inflammation swelling was evident in the model group but significantly decreased after erastin treatment; (b) micro-CT photographed the right hind foot to construct 3D images. Images showed significant bone destruction in all groups compared with the control group; (c) HE staining on the synovium of the right joint (100x).

The CIA and fer-1 animals showed more inflammation and significantly lower bone content than the control group got by 5 weeks of the experiment. Erastin could alleviate inflammation and damage of bone tissue. Collagen-induced immune response leads to the loss of normal synovial structure, proliferation of fibroblasts, and infiltration of lymphocytes in synovium. After 5 weeks of erastin intervention, proliferation of synovial cells and infiltration of lymphocytes were significantly reduced (Figure 9(a)–9(c)).

### 3.10. Ferroptosis Reduces Inflammation in Animal Model

**Figure 10:**
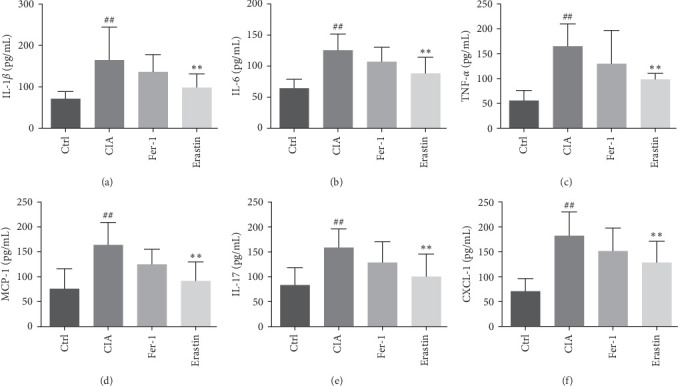
Ferroptosis regulated the production of IL-1*β* (a), IL-6 (b), TNF-*α* (c), IFN-*γ* (d), IL-17/IL-17 (e), and CXCL1 (f) in CIA animal plasma (^##^*P* < 0.01 vs. Ctrl group; *⁣*^*∗∗*^*P* < 0.01 vs. CIA group, *n* = 8).

The plasma from each group was supervised using ELISA, in which IL-1*β*, IL-6, TNF-*α*, IFN-*γ*, CXCL1, and IL-17/IL-17A were upregulated in CIA than the control group and downregulated in erastin (Figure 10(a)–10(f)). These phenomena indicated that to activate ferroptosis may potentially regulate these inflammatory factors.

## 4. Discussion

Human fibroblast-like synovial (FLS) cells in RA exhibit pathological hyperplasia and invasion, promoting tissue pan-angiogenesis, cartilage degradation, and the formation of bone erosion. In our research, it was found that FLS cells exhibit different phenotypes when stimulated by the ferroptosis activator erastin and the inhibitor fer-1. Ferroptosis activates inflammation and promotes the release of microglial proinflammatory factors in brain tissue [12]. It can also enhance autophagy activity and promote acute kidney injury [13]. Inhibitors of ferroptosis have been shown to have anti-inflammatory effects in experimental models of acute kidney injury, intracerebral hemorrhage, and neurodegenerative diseases [14–16]. TNF-*α* added in FLS cells, erastin, and TNF-*α* common significantly promoted FLS proliferation and migration, whereas fer-1 did the opposite. Mitochondrion, as an important organelle for redox reactions, may play a crucial part in the course of the iron death, despite it is controversial whether they are really involved in iron death [17, 18]. Mitochondrial dysfunction possibly leaded to ferroptosis. We examined mitochondrial membrane potential and ROS levels in human fibroblast-like synoviocyte cells and found that erastin induces an increase in mitochondrial membrane potential and promotes ROS release, whereas fer-1 does the opposite. Inhibition of VDAC prevents mitochondrial oxidative damage in hippocampal cells. Increased expression of TFR and DMT induces iron death activation in hereditary nephropathy. It is necessary to activate the IRP2-Iron-ROS axis to induce ferroptosis in hepatic stellate cells and to exert antifibrotic effects. It is clear that regulating the VDAC, DMT1, and IRP2 induces ferroptosis [19–22]. We found that erastin significantly promotes the expression of these proteins of VDAC, DMT1, TFR2, and IRP2 in human fibroblast-like synoviocyte cells, while FER-1 significantly inhibits them. Ferroptosis is due to the accumulation of cellular ROS exceeding the redox contents maintained by GSH and the phospholipid hydroperoxidases using GSH as a substrate. Ferroptosis was triggered by Erastin to inhibit the activity of cystine-glutamate antiporter (system Xc-), resulting in the depletion of cellular cysteine and GSH, which leads to the collapse of cellular redox homeostasis [23]. The increase in iron accumulation, excessive release of free radicals, and increased peroxides can induce ferroptosis. Levels of GSH, GPX4, Nrf2, Keap1, and ferritin in healthy subjects, active RA patients, and RA patients in remission were detected. The levels of GSH, GPX4, Nrf2, and Keap1 were distinctly reduced in active RA patients than in healthy ones, while the levels of these proteins were clearly higher in RA patients in remission than in active RA patients. Ferritin levels were significantly higher in patients during the acute phase of the disease than in healthy people and after treatment. It is suggested that iron death is crucial in the pathogenesis and remission of RA. We also observed mitochondria in human fibroblast-like synoviocytes cells using transmission electron microscopy, and it was found that erastin increased mitochondrial membrane density and number, while fer-1 inhibited mitochondrial growth. Ferroptosis is mainly controlled by the GPX4 [24]. We also found that erastin decreased the expression of GPX4, while fer-1 increased it. To our surprise, in animal experiments, we found that erastin significantly reduced the synovial inflammatory response and the expression of various inflammatory factors in the blood of CIA animals, whereas fer-1 did the opposite. In vivo or vitro, the ferroptosis was found to be nonlinear between cell models and animal models, and the results obtained in animal models were completely opposite to those obtained in human body and cells. According to the existing research, ROS plays a dual role in the human internal environment. A small amount of ROS will promote intercellular signal transmission and have positive significance on cell proliferation, immune signal transduction, and activation of functional proteins, but excessive ROS will increase the severity of diseases and cause oxidative imbalance in the body. We consider that the reasons may be when induced acute or chronic inflammation in the human body, ROS levels were positive feedback to increase and promote the development of the disease, which is related to the various proteins of antioxidant capacity in the body is constantly consumption. After drug treatment or ease, antioxidant levels self-repair and gradually returned to normal. In the cell model, giving a small amount of ferroptosis activator will stimulate the release of ROS, in turn, promote cell proliferation. The ferroptosis promotes inflammation subsidence of animal models. It may be associated with the recovery trend of the animals themselves; after all, animals cannot completely simulate the human body environment. When the animal's own recovery tendency is superimposed on the effect of ROS release and activation of the immune system to modulate the intensity of inflammation, a resolution of inflammation is induced instead. Therefore, in the basic research of RA or pharmacological research, animal models of phenotypic change cannot fully represent the pathological changes of human disease; it needs to be combined with a variety of models to multidimensional evaluation.

## 5. Conclusion

Therefore, we have reason to believe that activation of iron death can potentially promote the pathological development of RA, while amelioration of RA by inhibition of ferroptosis is limited to humans.

## Data Availability

Raw materials supporting the conclusions of this paper will be provided by the authors.
